# Herlyn-Werner-Wunderlich (HWW) Syndrome in an Adolescent

**DOI:** 10.7759/cureus.96788

**Published:** 2025-11-13

**Authors:** Joana Monteiro dos Santos Figueiredo, João Figueiredo

**Affiliations:** 1 Family Medicine, Unidade de Cuidados de Saúde Personalizados (UCSP) de Almeida, Unidade Local de Saúde (ULS) Guarda, Almeida, PRT; 2 General and Family Medicine, Unidade de Saúde Familiar (USF) Aliança, Unidade Local de Saúde (ULS) Entre Douro e Vouga, Oliveira de Azeméis, PRT

**Keywords:** adolescent gynecology, herlyn-werner-wunderlich syndrome, müllerian duct anomaly, renal agenesis, uterus didelphys

## Abstract

Herlyn-Werner-Wunderlich (HWW) syndrome, also known as obstructed hemivagina and ipsilateral renal anomaly (OHVIRA) syndrome, is a rare Müllerian duct anomaly characterized by the triad of uterus didelphys, obstructed hemivagina, and ipsilateral renal agenesis. Early diagnosis is essential to prevent complications and preserve reproductive potential.

We report the case of a 14-year-old female patient with significant dysmenorrhea and menstrual irregularities since menarche. The initial pelvic ultrasound was unremarkable. Nine months later, she presented repeatedly to the emergency department with left flank pain and vomiting. Further evaluation revealed the absence of the left kidney, uterus didelphys with an obstructed left hemi-uterus, hematocolpos, hematosalpinx, left ovarian endometrioma, and left renal agenesis on magnetic resonance imaging (MRI). Surgical management included laparoscopic hemihysterectomy of the left uterus and ipsilateral salpingectomy. Postoperatively, the patient was started on continuous progestogen-only therapy, with marked improvement in symptoms and quality of life.

HWWsyndrome typically manifests soon after menarche with cyclic pelvic pain, dysmenorrhea refractory to treatment, or pelvic mass. MRI is the gold standard for diagnosis, allowing precise anatomical characterization and guiding surgical planning. Without a functional or communicating obstructed hemicavity, hemihysterectomy with salpingectomy is an effective approach, preventing recurrence and reducing the risk of complications such as infection and endometriosis.

This case highlights the importance of maintaining a high index of suspicion for HWW syndrome in adolescents with refractory dysmenorrhea and associated renal anomalies. Early diagnosis and individualized surgical management can relieve symptoms, improve quality of life, and preserve reproductive potential.

## Introduction

Congenital malformations of the female genital tract represent a significant clinical challenge due to their potential impact on reproductive health, menstrual function, and the frequent diagnostic difficulties they pose. Müllerian duct anomalies (MDAs) occur in approximately 1-5% of the general female population, with higher prevalence, up to 13.3%, reported in women with recurrent pregnancy loss or infertility [1]. Among these anomalies, uterus didelphys is a rare entity, accounting for roughly 8% of MDAs and with an estimated prevalence of about 0.1-0.5% in the general population [2]. It results from a complete failure of Müllerian duct fusion during embryogenesis, leading to the development of two separate uterine cavities, usually with two distinct cervices, and often associated with a longitudinal vaginal septum.

Most cases remain asymptomatic until menarche, when patients may present with dysmenorrhea, cyclic pelvic pain, menstrual irregularities, or reproductive difficulties such as miscarriage or infertility [1]. Because the clinical presentation can be subtle or non-specific, imaging plays a central role in diagnosis and therapeutic planning. Techniques such as transvaginal ultrasound and magnetic resonance imaging (MRI) are essential for accurate anatomical characterization and to differentiate uterus didelphys from other MDAs, thereby guiding optimal management [3].

## Case presentation

A 14-year-old female patient presented to the family doctor with significant dysmenorrhea and menstrual irregularities since menarche at age 12. She had been using nonsteroidal anti-inflammatory drugs (NSAIDs) during the initial days of her cycle. Her medical history was unremarkable; she was on isotretinoin and combined oral contraceptive pills. Her first pelvic ultrasound showed normal uterine structure and a probable 30 mm follicular cyst in the ovary.

Nine months later, the patient attended the emergency department (ED) three times with left flank pain and vomiting; she remained afebrile and was diagnosed with possible endometrioma or hemorrhagic cyst and treated for a urinary tract infection.

**Figure 1:**
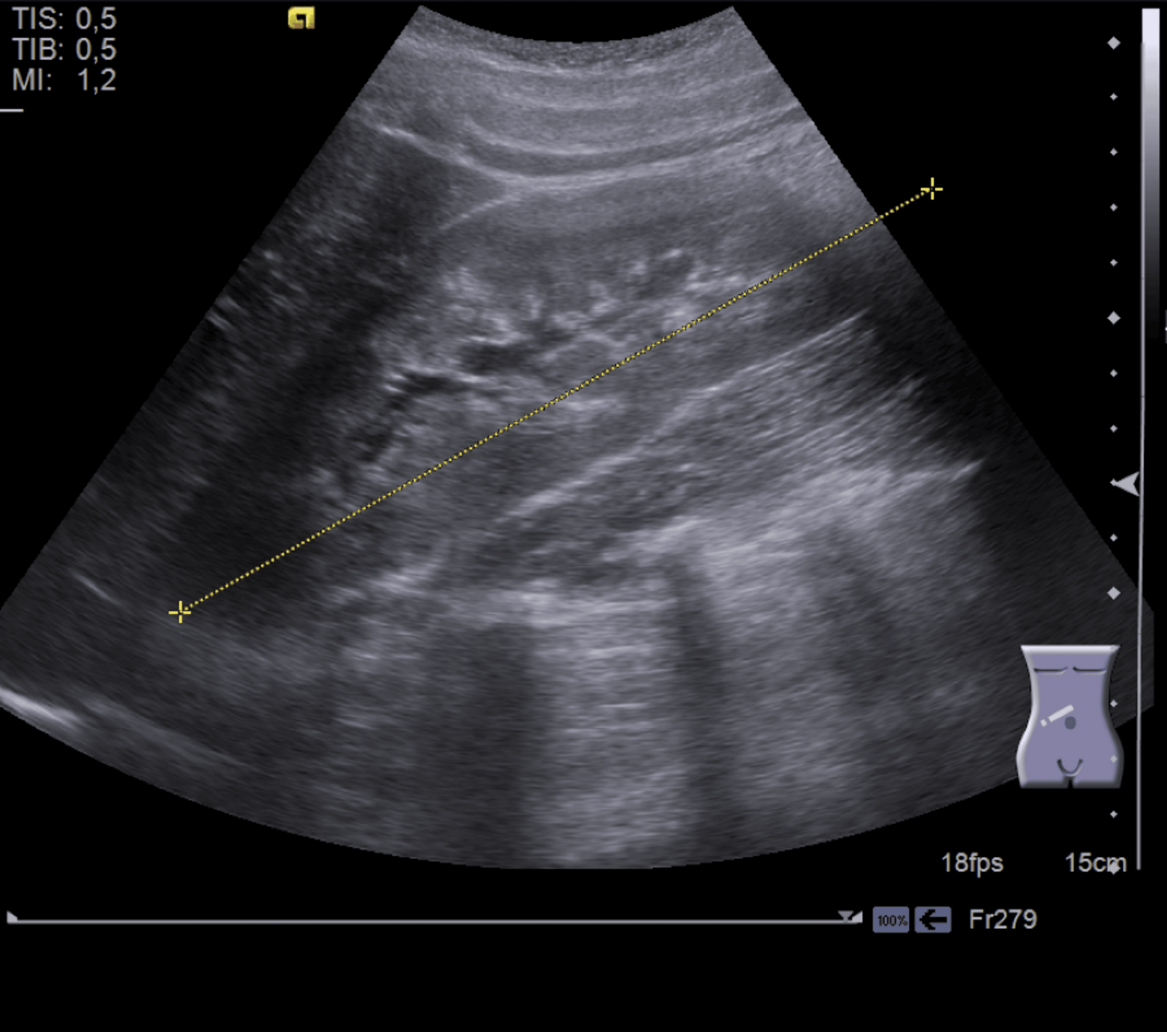
Longitudinal pelvic ultrasound Right kidney in normal topography, with compensatory-type morphology and increased volume, longitudinal diameter of 150 mm, regular contour, normal parenchyma-sinus differentiation, and preserved parenchymal thickness TIS: thermal index for soft tissue; TIB: thermal index for bone; MI: mechanical index

Persistent symptoms prompted referral to a tertiary hospital. Repeat pelvic and abdominal ultrasound revealed the absence of the left kidney with compensatory hypertrophy of the right, a suspected left hemi-uterus, adnexal cysts, and a normal right ovary (Figure 1).

**Figure 2:**
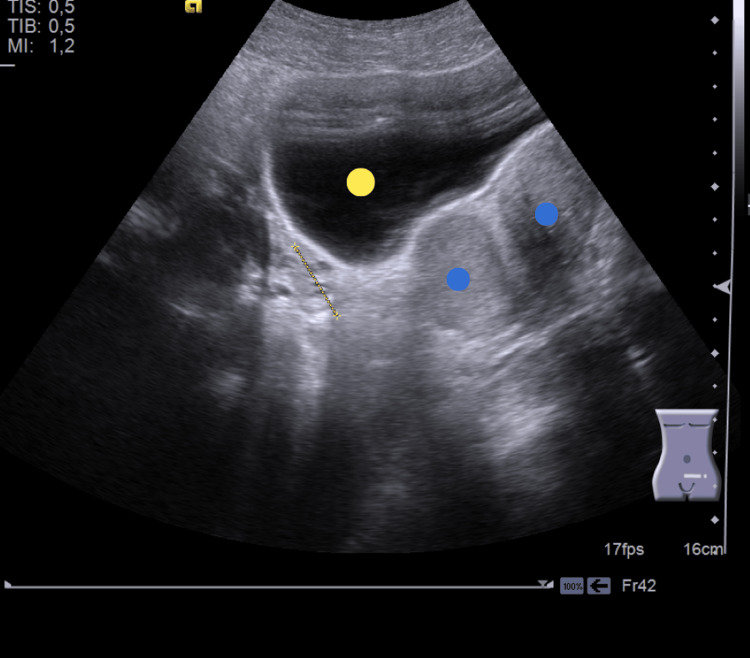
Pelvic ultrasound Blue dots: two uterine cavities. Yellow dot: urinary bladder. Yellow dashes on the left: one ovary TIS: thermal index for soft tissue; TIB: thermal index for bone; MI: mechanical index

Subsequent abdominal and transrectal ultrasound identified a normal right hemi‑uterus with hypoplastic endometrium (likely due to contraceptive use) and a distended left hemi‑uterus with hematometra and hematosalpinx, without hematocolpos (Figure 2).

**Figure 3:**
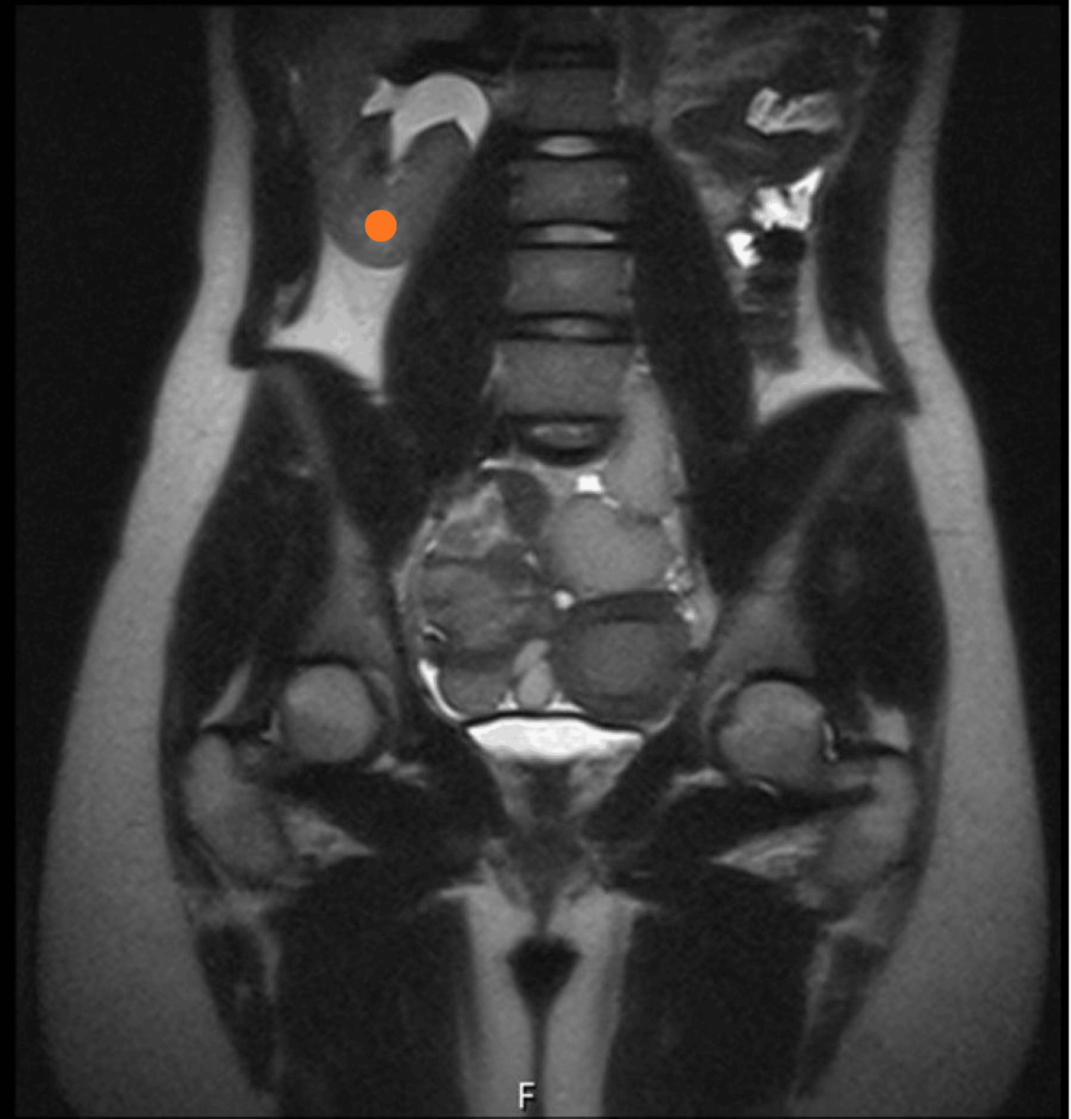
Coronal T2 MRI Orange dot: right compensatory kidney MRI: magnetic resonance imaging

**Figure 4:**
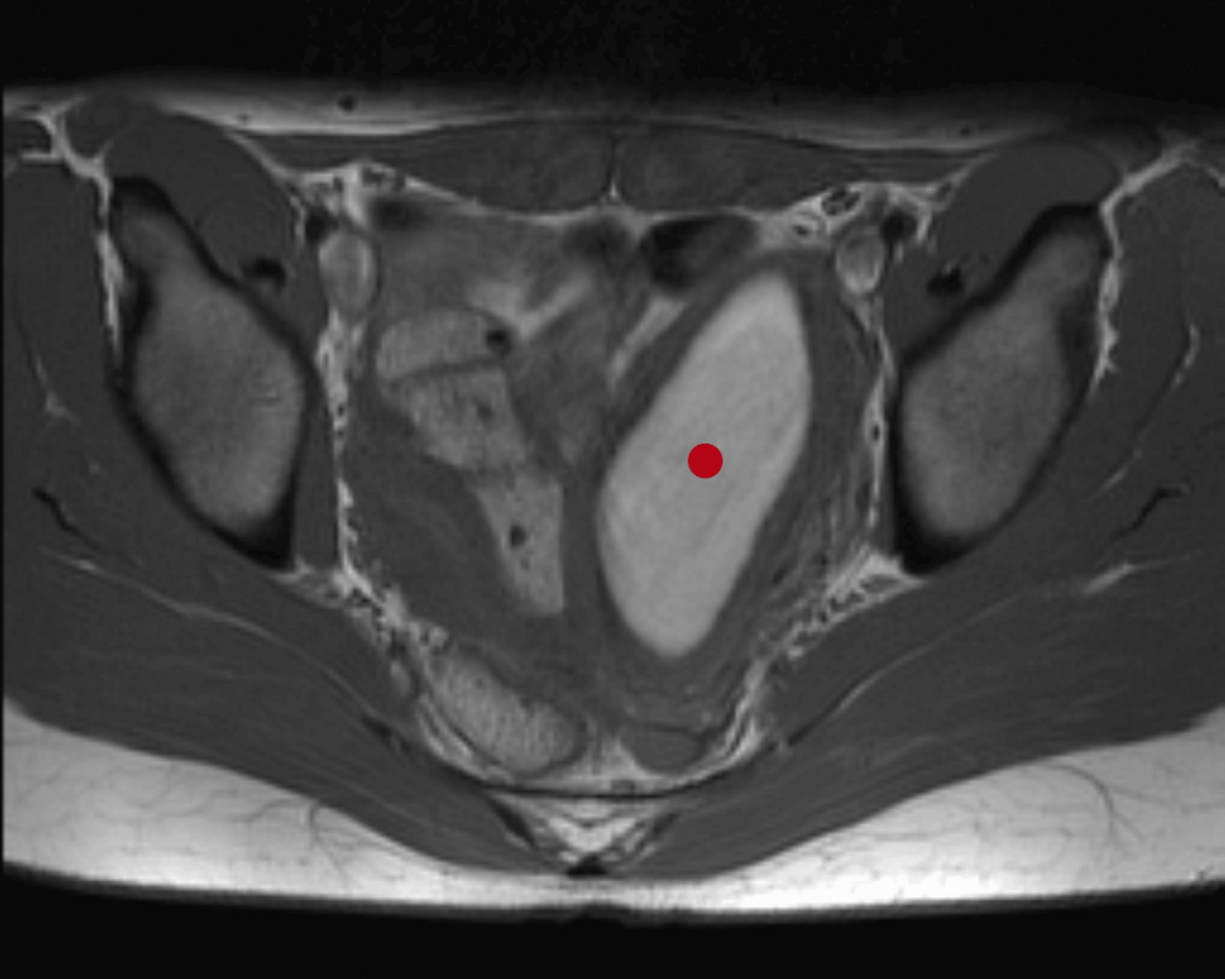
Axial T2 MRI Red dot: distended left uterine cavity MRI: magnetic resonance imaging

**Figure 5:**
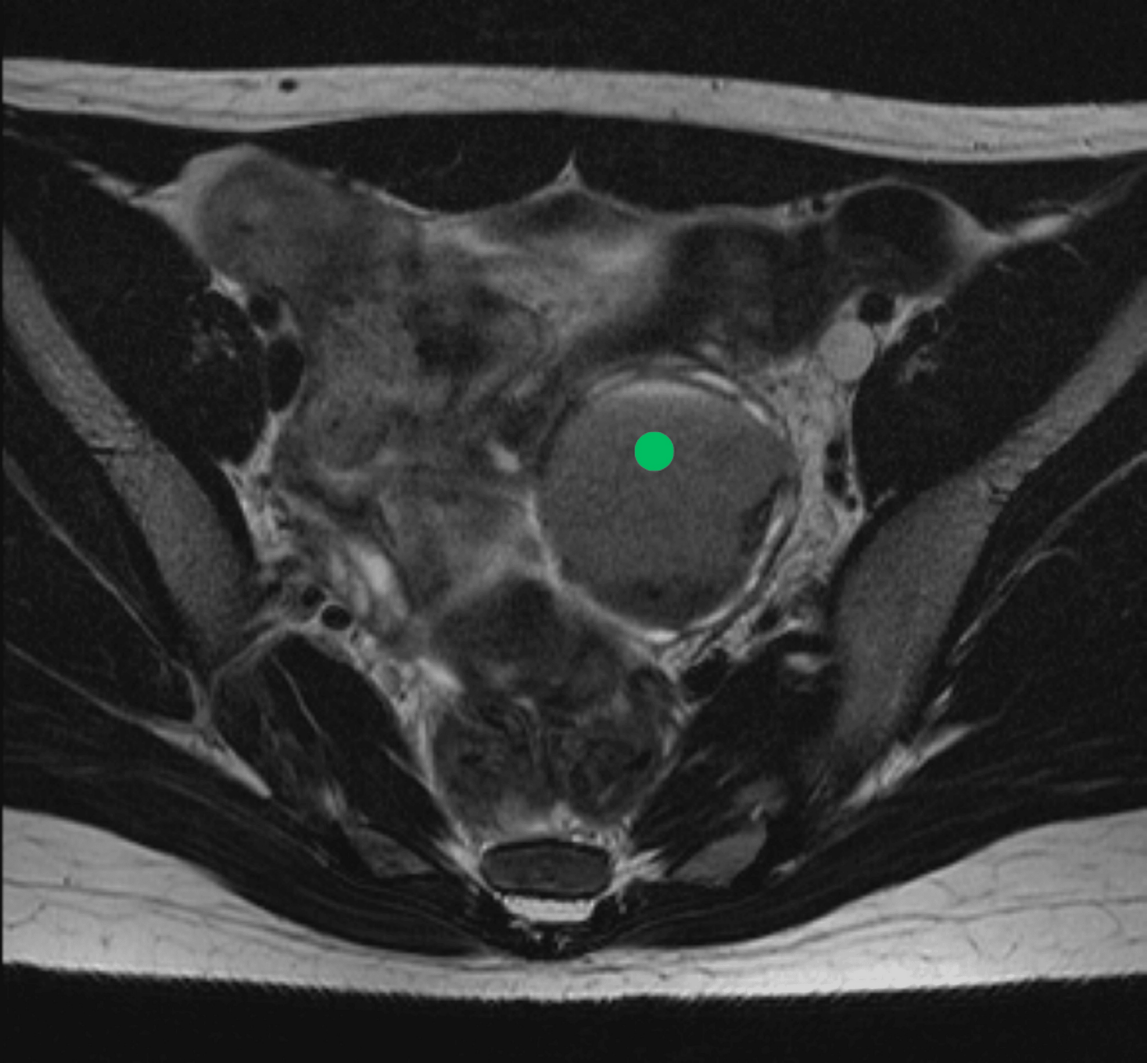
Axial T2 MRI Green dot: left ovarian endometrioma MRI: magnetic resonance imaging

Pelvic MRI confirmed uterus didelphys with an obstructed left hemi-uterus, hematocolpos, hematosalpinx, left ovarian endometrioma (45 mm), and left renal agenesis (Figure 3). The right uterine cavity communicated with the vagina and appeared normal (Figure 4 and Figure 5).

Surgical management comprised diagnostic laparoscopy, hysteroscopic evaluation (revealing no patent cervical canal on the left), laparoscopic hemihysterectomy of the left uterus, and ipsilateral salpingectomy. Postoperatively, the patient was started on a continuous progestogen-only pill (POP). She now has regular cycles of four to five days, normal flow, mild dysmenorrhea, and no intermenstrual or pelvic pain. Her quality of life has significantly improved, with no current gynecological complaints.

## Discussion

This case represents a classic but clinically challenging presentation of Herlyn-Werner-Wunderlich (HWW) syndrome, also referred to as obstructed hemivagina and ipsilateral renal anomaly (OHVIRA) syndrome, characterized by uterus didelphys, obstructed hemivagina or hemi-uterus, and ipsilateral renal agenesis [4]. In our patient, the initial presentation of severe dysmenorrhea and pelvic pain was consistent with the accumulation of menstrual blood in the obstructed left hemi-uterus, leading to hematometra and hematosalpinx, as well as the development of an ovarian endometrioma. Notably, hematocolpos, frequently described in this syndrome, was absent, highlighting the variability in anatomical obstruction patterns [3,5].

The diagnostic process was prolonged due to the non-specific early imaging findings. Initial pelvic ultrasound failed to detect the uterine anomaly, illustrating how standard transabdominal or even early transvaginal ultrasound may miss complex MDAs, especially when the non-communicating cavity is small or obscured by adjacent structures. The subsequent identification of left renal agenesis raised suspicion for an associated genital tract malformation, which was ultimately confirmed by MRI, a gold standard modality for precise anatomical characterization [5,6]. This reinforces that in adolescents with unexplained severe dysmenorrhea and renal anomalies, advanced imaging should be performed promptly.

The decision for laparoscopic hemihysterectomy with ipsilateral salpingectomy was based on the absence of a functional cervical canal on hysteroscopic evaluation and the risk of recurrent hematometra, endometriosis progression, and infection in a non-functional cavity [4,7]. While vaginal septum resection or vaginoplasty is often recommended in cases with a patent but obstructed hemivagina, definitive excision of a non-communicating hemi-uterus can prevent recurrence and protect the fertility potential of the contralateral side [7,8]. In our patient, postoperative symptom resolution and restoration of regular cycles align with reported outcomes for definitive surgical management [7].

Fertility outcomes in HWW/OHVIRA syndrome are generally favorable when the functioning uterus is preserved, with reported pregnancy rates ranging from 57% to 68% [7]. Although our patient is not yet seeking conception, the surgical approach chosen maximizes future reproductive potential while eliminating the source of pain and morbidity [9]. This case also emphasizes the importance of multidisciplinary care, including gynecology, radiology, and urology, for comprehensive evaluation and optimal long-term follow-up, especially given the lifelong implications of a solitary kidney [10].

## Conclusions

HWW/OHVIRA syndrome, while rare, should be suspected in adolescents presenting with severe dysmenorrhea refractory to treatment, especially when renal anomalies are identified. Early recognition and accurate anatomical delineation via MRI are critical for preventing complications and preserving fertility.

This case highlights that initial imaging may fail to reveal the diagnosis and advanced imaging should be pursued when clinical suspicion persists. In non-functional or non-communicating obstructed hemicavities, surgical intervention like hemihysterectomy with salpingectomy can offer symptom relief and protection of fertility. Timely diagnosis and intervention, coupled with multidisciplinary care, are essential for optimal reproductive outcomes in OHVIRA syndrome.
